# PSO-based parameter optimization of intuitionistic fuzzy generator for low-light image enhancement

**DOI:** 10.3389/frai.2026.1858540

**Published:** 2026-07-16

**Authors:** Uma Maheswari S., Jagatheswari S.

**Affiliations:** Department of Mathematics, School of Advanced Sciences, Vellore Institute of Technology, Vellore, Tamil Nadu, India

**Keywords:** BM3D denoising, gamma correction, intuitionistic fuzzy generator, low-light image enhancement, particle swarm optimization

## Abstract

Low-light images often suffer from reduced visibility, noise, and loss of structural details due to insufficient illumination and sensor limitations. These degradations affect both visual perception and downstream image analysis tasks. This paper presents a low-light image enhancement framework based on intuitionistic fuzzy generator (IFG) integrated with gamma correction and optimized using particle swarm optimization (PSO). As a preprocessing step, block-matching and 3D filtering (BM3D) are applied to suppress noise while preserving structural information. The IFG models uncertainty in pixel intensities to enable adaptive contrast enhancement, whereas gamma correction adjusts brightness levels. The enhancement parameters are optimized using PSO guided by dataset-specific objective functions, namely structural similarity (SSIM) for reference datasets and entropy-based optimization for no-reference scenarios where ground-truth images are unavailable. Experimental evaluations on standard benchmark datasets using both reference and no-reference image quality metrics indicate that the proposed framework achieves competitive enhancement performance with improved contrast and preservation of visually relevant image details. Although the computational cost is higher than that of feed-forward deep learning models, the framework is suitable for applications where training data are unavailable and interpretable parameter-adaptive enhancement is preferred.

## 1 Introduction

Image enhancement focuses on improving the visual quality and interpretability of images by highlighting important features while reducing degradations such as noise and low contrast. It plays an important role in areas including medical imaging, remote sensing, and forensic analysis, where accurate visual representation is necessary.

Images captured in low-light environments often suffer from poor illumination, sensor noise, and limited dynamic range. As a result, they usually exhibit reduced contrast, loss of detail, and uneven brightness distribution, which can negatively affect both visual perception and automated image analysis tasks such as segmentation, detection, and recognition. Therefore, an effective low-light enhancement method should improve visibility while preserving structural information and preventing excessive noise amplification.

Existing image enhancement methods can generally be classified into spatial-domain and frequency-domain techniques. Spatial-domain methods, including histogram equalization (Chen and Ramli, 2004), adaptive histogram equalization, gamma correction, and Retinex-based approaches, directly operate on pixel intensities and are computationally efficient. However, these methods may produce over-enhancement and visually unnatural artifacts in complex low-light scenes. Frequency-domain methods enhance images in transformed spaces such as the Fourier or wavelet domains, but they may introduce ringing artifacts and increase computational complexity.

More recent approaches incorporate adaptive strategies based on fuzzy logic, optimization techniques, and deep learning. Deep learning-based methods have achieved strong enhancement performance; however, they generally require large training datasets and high computational resources, which can limit their applicability in practical or data-scarce environments. In addition, their performance may decrease when the illumination conditions differ significantly from those encountered during training. In contrast, fuzzy logic-based methods provide an interpretable framework for handling uncertainty in image intensities. These methods transform pixel values into a fuzzy domain using membership functions and perform enhancement through fuzzy inference operations.

Despite the progress in low-light image enhancement, maintaining structural details while controlling noise and preserving natural illumination remains a challenging task. Many existing methods rely on fixed parameter settings or lack adaptability to different illumination conditions, whereas learning-based approaches may show limited robustness under unseen lighting distributions. These challenges motivate the development of a training-free and parameter-adaptive framework capable of enhancing low-light images while maintaining structural consistency and visually balanced illumination.

### 1.1 Core contributions

This work presents a low-light image enhancement framework based on the intuitionistic fuzzy generator (IFG), combined with block-matching and 3D filtering (BM3D) denoising and particle swarm optimization (PSO) for adaptive parameter tuning. The proposed method operates without the need for training data and adopts different optimization objectives for reference and no-reference datasets.

The major contributions of this work are summarized as follows:

- An adaptive IFG-based enhancement framework is proposed, where PSO is used to optimize the fuzzy control and gamma correction parameters according to image characteristics.
- A dataset-specific optimization strategy is introduced by employing SSIM for reference datasets and entropy-based objectives for no-reference datasets.
- A two-stage enhancement pipeline integrating BM3D denoising and IFG-based enhancement is developed to suppress noise while preserving important structural details.
- Experimental results on multiple benchmark datasets demonstrate competitive quantitative performance and visually balanced enhancement results.

The remainder of this article is organized as follows. Section 2 reviews the related literature. Section 3 presents the theoretical background, including fuzzy sets and their extensions, BM3D filtering, PSO, and gamma correction. Section 4 describes the proposed methodology, which integrates the IFG with gamma correction optimized using PSO. Section 5 presents the experimental results, including dataset descriptions, evaluation metrics, comparative analysis, and detailed discussions. Finally, Section 6 presents the conclusions and highlights future research directions.

## 2 Related work

A wide range of techniques has been developed for low-light image enhancement, including histogram-based methods, fuzzy logic approaches, optimization-based frameworks, Retinex models, and deep learning architectures. These methods mainly focus on improving visibility, contrast, and structural preservation under challenging illumination conditions.

Early low-light enhancement methods primarily relied on spatial-domain transformations such as histogram equalization, adaptive histogram equalization, gamma correction, and Retinex-based techniques. In Chen and Ramli (2004), a brightness-preserving histogram equalization method was introduced to improve image contrast while reducing over-enhancement artifacts. Similarly, Sharma and Verma (2016) proposed a fuzzy logic-based enhancement framework in the HSV color space using modified sigmoid and Gaussian membership functions to improve underexposed and overexposed regions. Although these methods are computationally efficient, they often suffer from issues such as over-enhancement, color distortion, and reduced adaptability in severely degraded low-light scenes.

Optimization-based approaches have also been explored to achieve adaptive parameter selection. In Bhandari and Maurya (2020), a brightness-preserving histogram equalization framework optimized through cuckoo search was proposed, where adaptive plateau limits help maintain natural brightness while avoiding excessive enhancement. A hybrid Differential Evolution and Cuckoo Search algorithm using entropy, edge, and contrast-based fitness functions for adaptive enhancement was presented in Suresh and Lal (2017). In applications such as remote sensing and medical imaging, optimization-driven techniques based on Gabor filtering, adaptive decomposition, and degradation-aware processing have shown promising performance under difficult imaging conditions (Asokan, 2023; Palani et al., 2026).

Retinex-based methods continue to be widely used because of their ability to separately model illumination and reflectance components. A noise-aware and detail-preserving Retinex framework combining variational decomposition, weighted illumination adjustment, and bilateral filtering was proposed in Veluchamy and Subramani (2025). Likewise, Subramani and Veluchamy (2023) introduced a bilateral tone-mapped gamma correction approach for enhancing poorly illuminated medical images. Despite their effectiveness in illumination correction, Retinex-based methods may produce halo artifacts, amplify noise, and introduce color inconsistencies under severe low-light conditions.

Fuzzy logic-based enhancement methods provide an interpretable and flexible framework for handling uncertainty in low-light images. An adaptive intuitionistic fuzzy enhancement approach combining global and local refinement strategies was presented in Joshua and Balasubramaniam (2025). In Veluchamy and Subramani (2020), a fuzzy dissimilarity contextual intensity transformation method integrated with gamma correction was developed for contrast enhancement. To improve uncertainty representation, an interval-valued intuitionistic fuzzy generator framework based on interval-valued membership and non-membership functions was introduced in Selvam and Sundaram (2025). Although these approaches improve contrast and structural visibility, many existing fuzzy-based frameworks still depend on fixed enhancement parameters and limited adaptive optimization strategies.

Noise suppression also plays an important role in low-light image enhancement. The BM3D denoising algorithm introduced in Dabov et al. (2007) groups similar image patches into 3D blocks and performs collaborative transform-domain filtering. BM3D remains one of the most effective denoising techniques for reducing noise while preserving structural details. Building on this concept, Yao et al. (2024) proposed a gradient-aware contrastive-adaptive framework for jointly improving illumination and structural preservation. Similarly, an intuitionistic fuzzy Retinex model designed for preserving image structures under low-light conditions was introduced in Ragavendirane and Dhanasekar (2025).

Recent progress in deep learning has significantly advanced low-light image enhancement research. CNN-based, GAN-based, transformer-based, and diffusion-based models have demonstrated strong capability in learning complex illumination transformations and perceptual representations. LE-GAN, an unsupervised GAN-based enhancement framework incorporating attention mechanisms and identity-invariant loss, was proposed in Fu et al. (2022). A gradient prior-guided network for maintaining structural consistency during enhancement was introduced in Lu et al. (2022).

Transformer-based methods have gained increasing attention because of their ability to model long-range contextual information. LA-Net, a biologically inspired enhancement framework that combines exposure correction and tone mapping through frequency decomposition, was proposed in Yang et al. (2023). Retinexformer, which integrates Retinex theory with transformer-based attention mechanisms for illumination correction and feature enhancement, was introduced in Cai et al. (2023). Likewise, Brateanu et al. (2025) proposed LYT-Net, a lightweight transformer-based model operating in the YUV color space to maintain illumination consistency while improving computational efficiency.

Diffusion-based frameworks have also shown promising enhancement capability in recent years. LLDiffusion, proposed in Wang et al. (2025), learns degradation representations for iterative low-light image refinement. Similarly, Xue et al. (2025) developed a CLIP-Fourier guided wavelet diffusion framework that combines frequency-domain priors with diffusion processes to improve perceptual contrast and structural visibility. Although these learning-based approaches achieve strong visual performance, they typically require large training datasets, high computational resources, and complex optimization procedures, which may limit their applicability in resource-constrained or data-scarce environments.

Perceptual image quality assessment has also become increasingly important in low-light enhancement studies. It was shown in Zhang et al. (2018) that deep feature-based perceptual similarity metrics correlate more closely with human visual perception than traditional measures such as PSNR and SSIM. Motivated by this observation, Demir and Kaplan (2023) proposed a low-light enhancement framework combining HSV transformation with a multi-scale Sharpening-Smoothing Image Filter (SSIF) to improve perceptual quality and visual consistency.

Overall, existing low-light enhancement approaches either rely on fixed parameter settings that limit adaptability under different illumination conditions or depend on data-driven models that require extensive training data and computational resources. While transformer and diffusion-based approaches provide strong enhancement capability, their deployment complexity may limit their use in training-free scenarios. In contrast, fuzzy and optimization-based methods offer interpretable and uncertainty-aware enhancement without supervised training; however, many existing fuzzy frameworks still employ fixed enhancement parameters and limited adaptive optimization. These limitations motivate the development of an adaptive framework that combines intuitionistic fuzzy modeling, optimization-based parameter selection, and denoising-aware preprocessing for robust low-light image enhancement.

### 2.1 Research gap and motivation

Although considerable progress has been made in low-light image enhancement, several challenges still remain. Many existing approaches depend on fixed parameter settings or supervised learning strategies, which limit their adaptability under different illumination conditions. In particular, most existing intuitionistic fuzzy generator (IFG)-based methods do not incorporate image-dependent parameter optimization, which can result in inconsistent enhancement quality and over-enhancement artifacts in varying low-light environments. Therefore, selecting enhancement parameters according to image characteristics is important for achieving stable and reliable performance across diverse lighting conditions. These limitations motivate the development of a training-free and noise-aware framework that combines intuitionistic fuzzy modeling with PSO-based parameter optimization and BM3D-guided denoising, enabling adaptive enhancement while preserving structural details and reducing noise amplification.

## 3 Preliminaries

### 3.1 Fuzzy set

The concept of fuzzy sets was introduced in Zadeh (1965). Let *X* denote the universe of discourse, where *x* ∈ *X*. A fuzzy set *V* ⊆ *X* is represented in Equation (1) as


$$\begin{array}{l} V=\left\{\left(x,\mu_{V}\left(x\right)\right)|x\in X\right\}, \end{array}$$ (1)

where μ_*V*_ : *X* → [0, 1] is the membership function of *V*.

### 3.2 Intuitionistic fuzzy set

An intuitionistic fuzzy set V^*^ in *X* (Atanassov, 1999; Chaira, 2019) is defined in Equation (2) as


$$\begin{array}{l} V^{*}=\left\{\langle x,\mu_{V^{*}}\left(x\right),\nu_{V^{*}}\left(x\right)\rangle |x\in X\right\}, \end{array}$$ (2)

where $\mu_{V^{*}}\left(x\right):X\to \left[0,1\right]$ and $\nu_{V^{*}}\left(x\right):X\to \left[0,1\right]$ denote the degrees of membership and non-membership of *x* ∈ *X* in V^*^, respectively, subject to the condition given in Equation (3)


$$\begin{array}{l} 0\leq \mu_{V^{*}}\left(x\right)+\nu_{V^{*}}\left(x\right)\leq 1. \end{array}$$ (3)

The hesitation degree of *x* ∈ *X* with respect to V^*^ is defined in Equation (4) as


$$\begin{array}{l} \pi_{V^{*}}\left(x\right)=1-\mu_{V^{*}}\left(x\right)-\nu_{V^{*}}\left(x\right), \end{array}$$ (4)

where $\pi_{V^{*}}\left(x\right)\in \left[0,1\right].$ Thus, for every *x* ∈ *X* $\mu_{V^{*}}\left(x\right)+\nu_{V^{*}}\left(x\right)+\pi_{V^{*}}\left(x\right)=1$ is satisfied. The mathematical symbols and notations used throughout this study are summarized in Table 1.

**Table 1:** Summary of mathematical notation.

Symbol	Description
*I*	Input low-light image
χ_*ij*_	Pixel intensity at position (*i, j*)
${\hat{\chi }}_{ij}$	Reconstructed pixel intensity after defuzzification
χ_max_, χ_min_	Maximum and minimum pixel values of image *I*
μ_Ī_(χ_*ij*_)	Normalized pixel intensity, μ_Ī_(χ_*ij*_) ∈ [0, 1]
$\mu_{\bar{IFI}}\left(\chi_{ij}\right)$	Degree of membership of the Intuitionistic Fuzzy Image
$\nu_{\bar{IFI}}\left(\chi_{ij}\right)$	Degree of non-membership of the Intuitionistic Fuzzy Image
$\pi_{\bar{IFI}}\left(\chi_{ij}\right)$	Hesitation degree of the Intuitionistic Fuzzy Image
*IFI*(χ_*ij*_)	Enhanced pixel value generated by the Intuitionistic Fuzzy Generator
α	Fuzzy control parameter (governs IFG transformation)
γ	Gamma correction parameter
*f*(·)	Auxiliary increasing function used in non-membership derivation
${\bar{\mu }}_{i},\;{\bar{\mu }}_{o}$	Mean intensities of original and enhanced images
$\sigma_{i}^{2},\;\sigma_{o}^{2}$	Variances of original and enhanced images
σ_*io*_	Covariance between original and enhanced images
*I*(*x, y*)	Pixel intensity of original image at location (*x, y*)
*T*(*x, y*)	Pixel intensity of enhanced image at location (*x, y*)
*w*	PSO inertia weight
*c*_1_, *c*_2_	PSO cognitive and social acceleration coefficients
*r*_1_, *r*_2_	Uniformly distributed random numbers in [0, 1]
*b*_*j*_	Personal best position of particle *j*
*g*	Global best position across the swarm

### 3.3 Block-matching and 3D filtering (BM3D)

BM3D (Dabov et al., 2007) is a powerful denoising technique that leverages the repetitive structures within an image and applies collaborative filtering in a 3D transform space. Unlike conventional local filters, BM3D begins by locating and grouping similar patches from different regions of the image through block matching. These matched patches are then combined into a 3D group and subjected to a linear transform, such as the discrete cosine transform (DCT). In the transformed domain, the underlying image structures appear sparse, while the noise is spread more uniformly. By applying thresholding or Wiener filtering, the noise components are effectively reduced. The filtered blocks are then inversely transformed and merged back into the image through weighted averaging. This method enables BM3D to reliably eliminate Gaussian noise while retaining essential image structures, textures, and fine details.

### 3.4 Particle swarm optimization

Particle swarm optimization (PSO) (Kennedy and Eberhart, 1995) is a population-based optimization algorithm inspired by the social behavior of bird flocking. It is widely used for solving complex optimization problems where analytical optimization methods are difficult to apply. In PSO, each individual in the population, called a particle, represents a potential solution to the optimization problem. These particles move through the solution space by updating their velocities and positions based on their own experience (personal best) and that of neighboring particles or the entire swarm (global best). The algorithm initializes a swarm of particles with random positions and velocities. Each particle's fitness is then evaluated using an objective function to determine the quality of the solution. Based on this evaluation, each particle updates its personal best position, and the global best across the swarm is tracked accordingly. The velocity and position of each particle are updated according to Equations (5) and (6):


$$\begin{array}{l} v_{j}\left(t+1\right)=w\cdot v_{j}\left(t\right)+c_{1}\cdot r_{1}\cdot \left(b_{j}-x_{j}\left(t\right)\right)+c_{2}\cdot r_{2}\cdot \left(g-x_{j}\left(t\right)\right), \end{array}$$ (5)

$$\begin{array}{l} x_{j}\left(t+1\right)=x_{j}\left(t\right)+v_{j}\left(t+1\right), \end{array}$$ (6)

where *x*_*j*_(*t*) and *v*_*j*_(*t*) denote the position and velocity of the *j*-th particle at iteration *t*, respectively. In these equations, *w* denotes the inertia weight, *c*_1_ and *c*_2_ are acceleration coefficients, and *r*_1_ and *r*_2_ are random numbers in [0, 1]. Here, *b*_*j*_ is the personal best, and *g* is the global best. These updates help guide the swarm toward optimal solutions by striking a balance between exploration and exploitation. The process is repeated iteratively until convergence is achieved or the maximum iteration count is reached.

### 3.5 Gamma correction

Gamma correction (Mishra et al., 2024; Rahman et al., 2016) is an image enhancement technique that adjusts the gamma value based on image characteristics rather than using a fixed setting. It is a non-linear transformation that modifies pixel intensities to control image brightness and contrast and is commonly expressed in Equation (7) as


$$\begin{array}{l} I_{\text{out}}\left(i,j\right)={\left(I_{\text{in}}\left(i,j\right)\right)}^{\gamma }. \end{array}$$ (7)

In adaptive gamma correction, the gamma value is determined by analyzing factors such as the image histogram and illumination level or by using optimization techniques. This adaptability allows the method to enhance underexposed or overexposed regions more effectively, thereby improving overall visibility while preserving details in both dark and bright areas.

## 4 Proposed intuitionistic fuzzy method

The proposed method enhances low-light images using an intuitionistic fuzzy generator (IFG)-based framework. Initially, the input low-light image *I* is transformed into a fuzzy domain through a fuzzification process. In image processing, fuzzification converts a crisp image with pixel intensities in the range [0, 255] into normalized membership values within [0, 1]. The membership function of the input image is defined in Equation (8) as


$$\begin{array}{l} \mu_{Ī}\left(\chi_{ij}\right)=\frac{\chi_{ij}-\chi_{\min}}{\chi_{\max}-\chi_{\min}}, \end{array}$$ (8)

where χ_*ij*_ represents the luminance value of the (*i, j*)-th pixel, and χ_max_ and χ_min_ denote the maximum and minimum pixel values of image *I*, respectively.

To extend the fuzzy representation into an intuitionistic fuzzy framework, an intuitionistic fuzzy generator (IFG) is employed using (Chaira, 2014). The IFG is defined as a mapping ψ:[0, 1] → [0, 1] that satisfies the condition ψ(*x*) ≤ (1−*x*), ∀*x* ∈ [0, 1].

Let $\bar{N}:\left[0,1\right]\to \left[0,1\right]$ be an increasing involutive fuzzy complement function. The complement function satisfies the involutive fuzzy complement property if there exists a continuous and strictly increasing generator function *f* such that *f*(0) = 0. Accordingly, the fuzzy complement is expressed in Equation (9) as


$$\begin{array}{l} \bar{N}\left(\mu_{Ī}\left(\chi_{ij}\right)\right)=f^{-1}(f\left(1\right)-f\left(\mu_{Ī}\left(\chi_{ij}\right)\right)). \end{array}$$ (9)

The proposed IFG employs the new increasing generator function given in Equation (10):


$$\begin{array}{l} f\left(\mu_{Ī}\left(\chi_{ij}\right)\right)=\frac{1}{\alpha }\ln\;(1+\left(2+\alpha \right)\mu_{Ī}\left(\chi_{ij}\right)), \end{array}$$ (10)

The parameter α controls the shape of the intuitionistic fuzzy generator and regulates the transformation strength of the membership and non-membership functions. Smaller values of α produce smoother intensity transitions and moderate enhancement behavior, whereas larger values increase enhancement strength by amplifying low-intensity membership values more aggressively. Hence, α provides adaptive control between contrast enhancement and detail preservation in low-light image regions.

The generator function satisfies the boundary conditions given in Equation (11)


$$\begin{array}{l} f\left(0\right)=\frac{1}{\alpha }\ln(1+\left(2+\alpha \right)\cdot 0)=0,\;\text{and}\\ f\left(1\right)=\frac{1}{\alpha }\ln(1+\left(2+\alpha \right)\cdot 1)\\ \;=\frac{1}{\alpha }\ln\left(3+\alpha \right). \end{array}$$ (11)

The inverse of *f*(μ_Ī_(χ_*ij*_)) is given by:


$$\begin{array}{l} f^{-1}\left(\mu_{Ī}\left(\chi_{ij}\right)\right)=\frac{e^{\alpha \mu_{Ī}\left(\chi_{ij}\right)}-1}{2+\alpha }. \end{array}$$ (12)

Substituting Equations (10) and (11) into Equation (9) and simplifying yields Equation (13)


$$\begin{array}{l} \bar{N}\left(\mu_{Ī}\left(\chi_{ij}\right)\right)=f^{-1}\left[\frac{1}{\alpha }\ln\left(\frac{3+\alpha }{1+\left(2+\alpha \right)\mu_{Ī}\left(\chi_{ij}\right)}\right)\right]. \end{array}$$ (13)

Using the inverse function in Equation (12), we obtain Equation (14)


$$\begin{array}{l} \bar{N}\left(\mu_{Ī}\left(\chi_{ij}\right)\right)=\frac{e^{\ln\left(\frac{3+\alpha }{1+\left(2+\alpha \right)\mu_{Ī}\left(\chi_{ij}\right)}\right)}-1}{2+\alpha }, \end{array}$$ (14)

Simplifying Equation (14), we obtain Equation (15)


$$\begin{array}{l} \bar{N}\left(\mu_{Ī}\left(\chi_{ij}\right)\right)=\frac{1-\mu_{Ī}\left(\chi_{ij}\right)}{1+\left(2+\alpha \right)\mu_{Ī}\left(\chi_{ij}\right)},\;\alpha >0. \end{array}$$ (15)

The complement function satisfies the fuzzy complement boundary conditions


$$\bar{N}\left(0\right)=\frac{1-0}{1+\left(2+\alpha \right)\left(0\right)}=\frac{1}{1}=1.$$

$$\bar{N}\left(1\right)=\frac{1-1}{1+\left(2+\alpha \right)\left(1\right)}=\frac{0}{3+\alpha }=0.$$

Using the proposed IFG, the membership degree of the intuitionistic fuzzy image (IFI) is defined in Equation (16) as


$${\bar{\mu }}_{\bar{IFI}}\left(\chi_{ij}\right)=1-\bar{N}\left(\mu_{Ī}\left(\chi_{ij}\right)\right)$$

which gives


$$\begin{array}{l} \mu_{\bar{IFI}}\left(\chi_{ij}\right)=1-\frac{1-\mu_{Ī}\left(\chi_{ij}\right)}{1+\left(2+\alpha \right)\mu_{Ī}\left(\chi_{ij}\right)}\\ \mu_{\bar{IFI}}\left(\chi_{ij}\right)=\frac{\left(3+\alpha \right)\mu_{Ī}\left(\chi_{ij}\right)}{1+\left(2+\alpha \right)\mu_{Ī}\left(\chi_{ij}\right)}. \end{array}$$ (16)

Similarly, the non-membership degree of IFI is expressed in Equation (17) as


$$\begin{array}{l} \nu_{\bar{IFI}}\left(\chi_{ij}\right)=\frac{1-\mu_{\bar{IFI}}\left(\chi_{ij}\right)}{1+\left(2+\alpha \right)\mu_{\bar{IFI}}\left(\chi_{ij}\right)}, \end{array}$$ (17)

Substituting Equation (16) into Equation (17) yields Equation (18)


$$\begin{array}{l} \nu_{\bar{IFI}}\left(\chi_{ij}\right)=\frac{1-\mu_{\bar{I}}\left(\chi_{ij}\right)}{1+\left(\alpha^{2}+6\alpha +8\right)\mu_{\bar{I}}\left(\chi_{ij}\right)}, \end{array}$$ (18)

The hesitation degree is then computed using the intuitionistic fuzzy relation given in Equation (19)


$$\begin{array}{l} \pi_{\bar{IFI}}\left(\chi_{ij}\right)=1-\mu_{\bar{IFI}}\left(\chi_{ij}\right)-\nu_{\bar{IFI}}\left(\chi_{ij}\right). \end{array}$$ (19)

In the proposed IFG framework, fuzzy uncertainty is quantified using the hesitation degree, which represents the indeterminacy between the membership and non-membership functions. Finally, the enhanced intuitionistic fuzzy image is obtained using Equation (20)


$$\begin{array}{l} IFI\left(\chi_{ij}\right)=\mu_{\bar{IFI}}\left(\chi_{ij}\right)+\pi_{\bar{IFI}}\left(\chi_{ij}\right), \end{array}$$ (20)

where *IFI*(χ_*ij*_) denotes the enhanced intuitionistic fuzzy pixel value.

### 4.1 Proposed methodology

The proposed framework begins with a noise reduction stage in which the low-light input image is processed using BM3D filtering to suppress noise amplified under poor illumination conditions. BM3D effectively removes noise while preserving edges and fine image details.

Applying BM3D before fuzzy enhancement improves the reliability of the enhancement process. The proposed intuitionistic fuzzy generator (IFG) computes membership, non-membership, and hesitation degrees from local intensity variations. In the presence of noise, these variations may be misinterpreted as meaningful structures, leading to inaccurate fuzzy memberships and amplification of noise artifacts during enhancement. Pre-denoising ensures that the fuzzy components are mainly derived from actual image structures, improving the stability of fuzzy membership estimation and parameter optimization.

Applying BM3D after enhancement is less effective because enhancement may transform noise into signal-dependent artifacts that are difficult to separate from structural details. Although pre-denoising may slightly smooth fine textures, it reduces noise propagation and improves the structural consistency of the enhanced image.

After denoising, the image is enhanced using the IFG-based model combined with gamma correction for luminance refinement. The enhancement process is controlled by the fuzzy control parameter α and the gamma correction parameter γ, which are optimized using PSO.

Initially, a swarm of particles is generated, where each particle represents a candidate parameter pair (α, γ) with corresponding velocities. Each particle applies the IFG-based enhancement to produce an enhanced image. For reference datasets, the enhancement quality is evaluated using the Structural Similarity Index (SSIM), while entropy is used as an optimization objective for no-reference datasets to improve information distribution and contrast representation.

Based on the fitness values, the personal best (pbest) and global best (gbest) solutions are updated. The particle positions and velocities are iteratively refined using standard PSO update equations to obtain the optimal parameter combination. After convergence, the enhanced image corresponding to the global best solution is selected as the final output.

### 4.2 Parameter setting

The PSO algorithm employs a swarm size of *N* = 6 particles with a maximum of 8 iterations. The inertia weight is fixed at *w* = 0.6 to balance exploration and exploitation, while the cognitive and social coefficients are set to *c*_1_ = 1.3 and *c*_2_ = 1.3, respectively. These parameters were selected through preliminary experiments and sensitivity analysis to ensure stable convergence and avoid premature stagnation. As shown in Table 2, smaller acceleration coefficients weakened optimization performance, whereas larger coefficients increased runtime and reduced convergence stability. Similarly, higher inertia weights degraded enhancement consistency due to unstable particle movement. Hence, the selected PSO parameters provide a balance between enhancement quality, convergence stability, and computational cost.

**Table 2:** Sensitivity analysis of PSO control parameters (*w*, *c*_1_, and *c*_2_) on the LOL dataset.

*w*	*c*_1_	*c*_2_	Avg. SSIM	Avg. PSNR (dB)	Avg. Runtime (s)
0.4	1.3	1.3	0.8019	23.9186	11.72
0.6	1.3	1.3	0.8039	23.8547	15.73
0.8	1.3	1.3	0.7966	23.5936	15.70
0.6	1.0	1.0	0.7831	22.0527	15.05
0.6	1.8	1.8	0.8045	23.8483	17.86

Initially, particles are randomly initialized within predefined bounds, where each particle represents a candidate parameter pair (α, γ). The search space is defined as α ∈ [0.1, 1.0] and γ ∈ [0.5, 2.5], where α controls the intuitionistic fuzzy generator (IFG) for contrast enhancement and γ regulates non-linear brightness adjustment. These bounds were selected based on preliminary experiments on representative low-light images. Values of α < 0.1 produced weak enhancement, while α>1.0 caused over-enhancement and noise amplification. Similarly, γ < 0.5 resulted in insufficient brightness correction, whereas γ>2.5 introduced saturation and detail loss. Therefore, the selected ranges provide stable enhancement performance while minimizing visual artifacts and optimization instability.

After BM3D-based denoising, the proposed framework jointly optimizes α and γ for contrast and brightness enhancement. Unlike fixed gamma correction methods, PSO adaptively determines image-dependent parameter values, while the IFG models local intensity uncertainty using membership, non-membership, and hesitation degrees to preserve brightness and structural details.

For reference-based datasets such as LOL, the SSIM is used as the optimization objective because it evaluates luminance, contrast, and structural similarity with respect to the ground-truth image. For no-reference datasets such as DICM, NPE, LIME, and MEF, entropy is employed as an optimization objective to improve information distribution and contrast representation.

The optimization process terminates when either the maximum number of iterations is reached or the absolute difference between two consecutive global best fitness values becomes smaller than 10^−5^. Since only two bounded parameters are optimized, a small swarm size and limited iterations are sufficient to achieve stable convergence with low computational complexity. For each test image, PSO optimization is executed once, and the final enhanced images are evaluated using SSIM and PSNR, with average results reported over the dataset.

### 4.3 Defuzzification

Defuzzification (Radhika and Parvathi, 2016) is the process of converting a fuzzy value into a crisp value, and the same principle applies to fuzzified images. The defuzzification of a fuzzified image is expressed in Equation (21) as:


$$\begin{array}{l} {\hat{\chi }}_{ij}=\mu_{Ī}\left(\chi_{ij}\right)\cdot \left(\chi_{\max}-\chi_{\min}\right)+\chi_{\min}, \end{array}$$ (21)

where ${\hat{\chi }}_{ij}$ denotes the reconstructed pixel intensity obtained after defuzzification. For the intuitionistic fuzzy image (IFI), the defuzzification equation is modified as given in Equation (22):


$$\begin{array}{l} {\hat{\chi }}_{ij}=IFI\left(\chi_{ij}\right)\cdot \left(\chi_{\max}-\chi_{\min}\right)+\chi_{\min}. \end{array}$$ (22)

## 5 Experimental analysis

This section describes the experimental setup, datasets, evaluation metrics, implementation details, and comparison methods used to assess the proposed low-light enhancement framework.

The proposed method was implemented in MATLAB R2023a with the Image Processing Toolbox on a Windows 10 system equipped with an Intel(R) Core(TM) i3-1005G1 CPU operating at 1.20 GHz, 4 GB RAM, and a 1.14 TB hard disk. Experiments were conducted using the reference-based LOL (Wei et al., 2018) dataset and the no-reference DICM (Lee et al., 2013), LIME (Guo et al., 2016), MEF (Lee et al., 2011), and NPE (Wang et al., 2013) datasets. The complete workflow of the proposed IFG-based image enhancement framework is summarized in Algorithm 1.

Algorithm 1 Pseudo-code of proposed IFG-based image enhancement framework. 
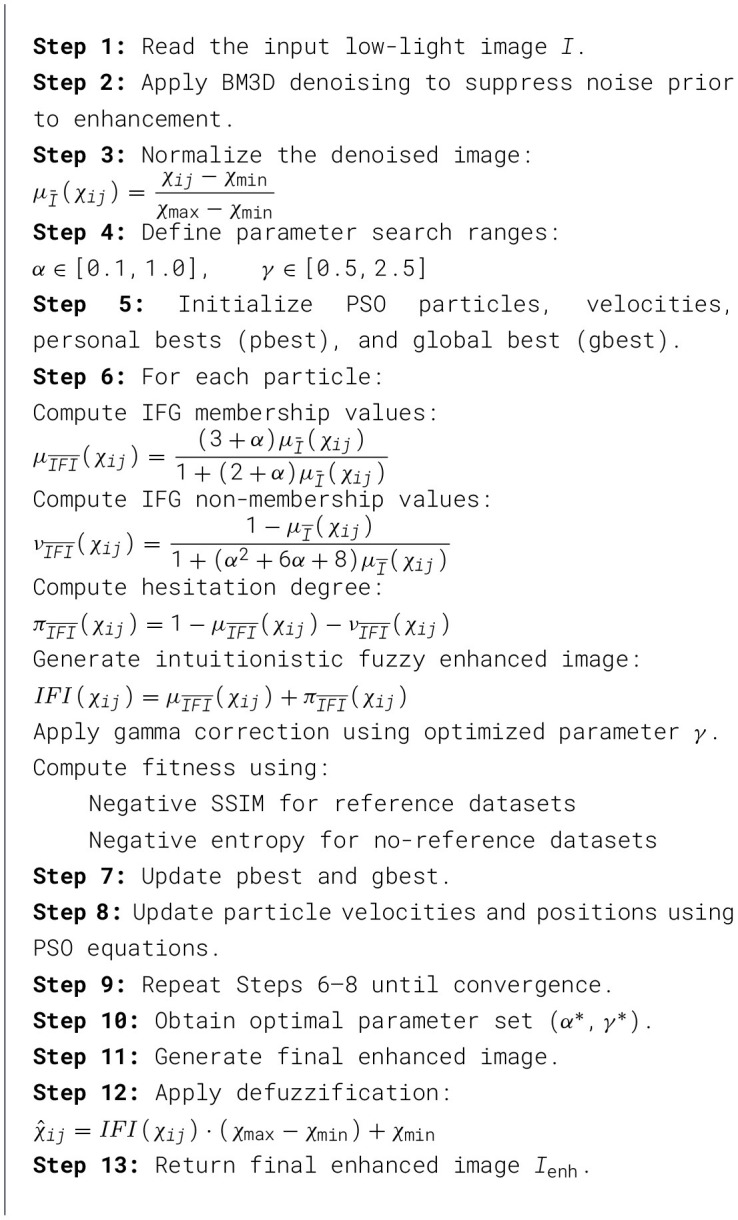

For BM3D preprocessing, the noise standard deviation was fixed at σ = 2/255 using the normal profile configuration. BM3D filtering was applied prior to fuzzy enhancement to reduce noise while preserving edge and structural information.

**Figure 1:**
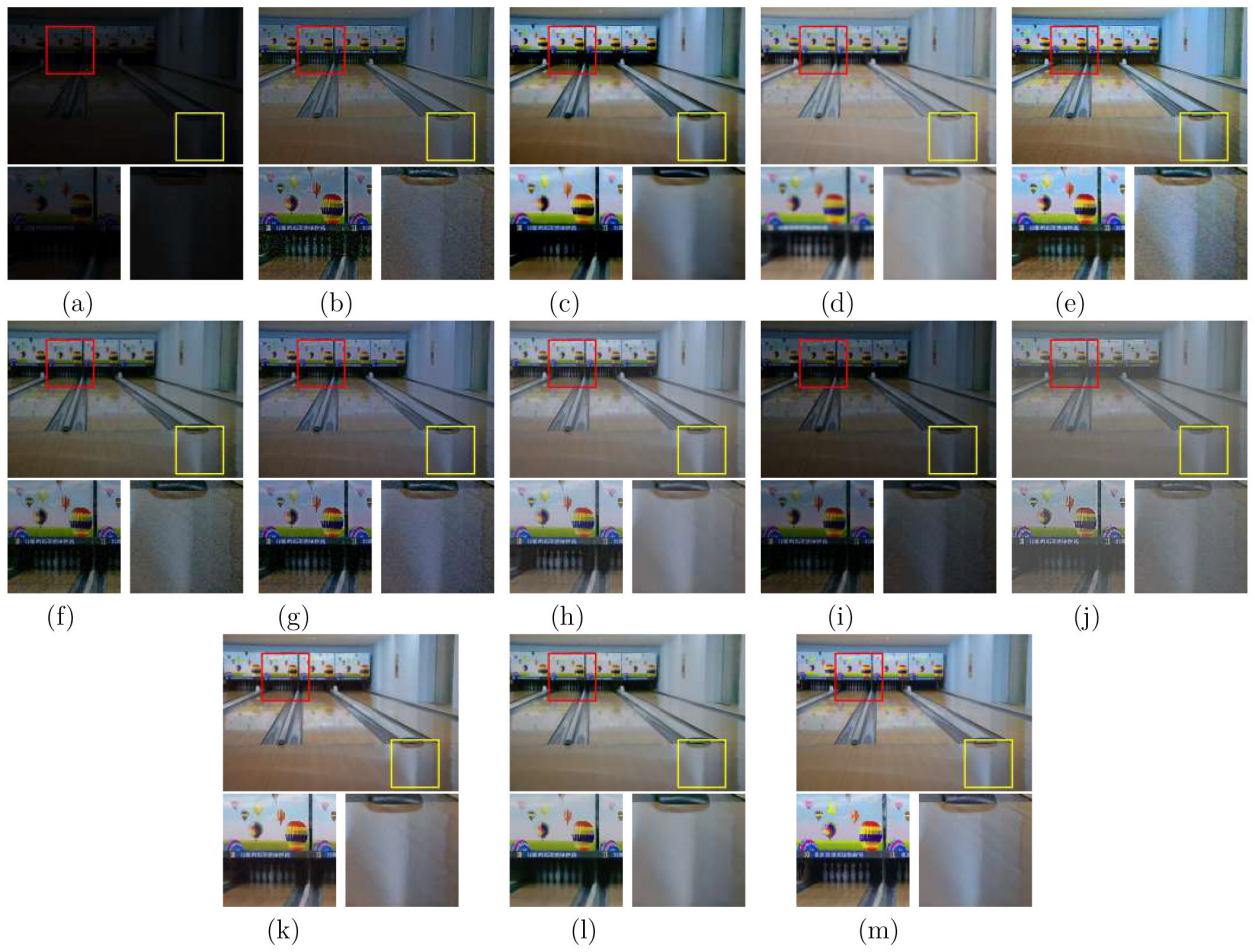
Qualitative comparison for Input-1 on the LOL dataset. Highlighted regions indicate zoomed-in comparisons for visual detail assessment. **(a)** Input-1, **(b)** LIME, **(c)** MBLLEN, **(d)** KIND, **(e)** SSIENeT, **(f)** Zero-DCE, **(g)** Zero-DCE++, **(h)** URetinex, **(i)** Brain, **(j)** NSGA, **(k)** LYT-Net, **(l)** Ours-1, **(m)** GT-1.

**Figure 2:**
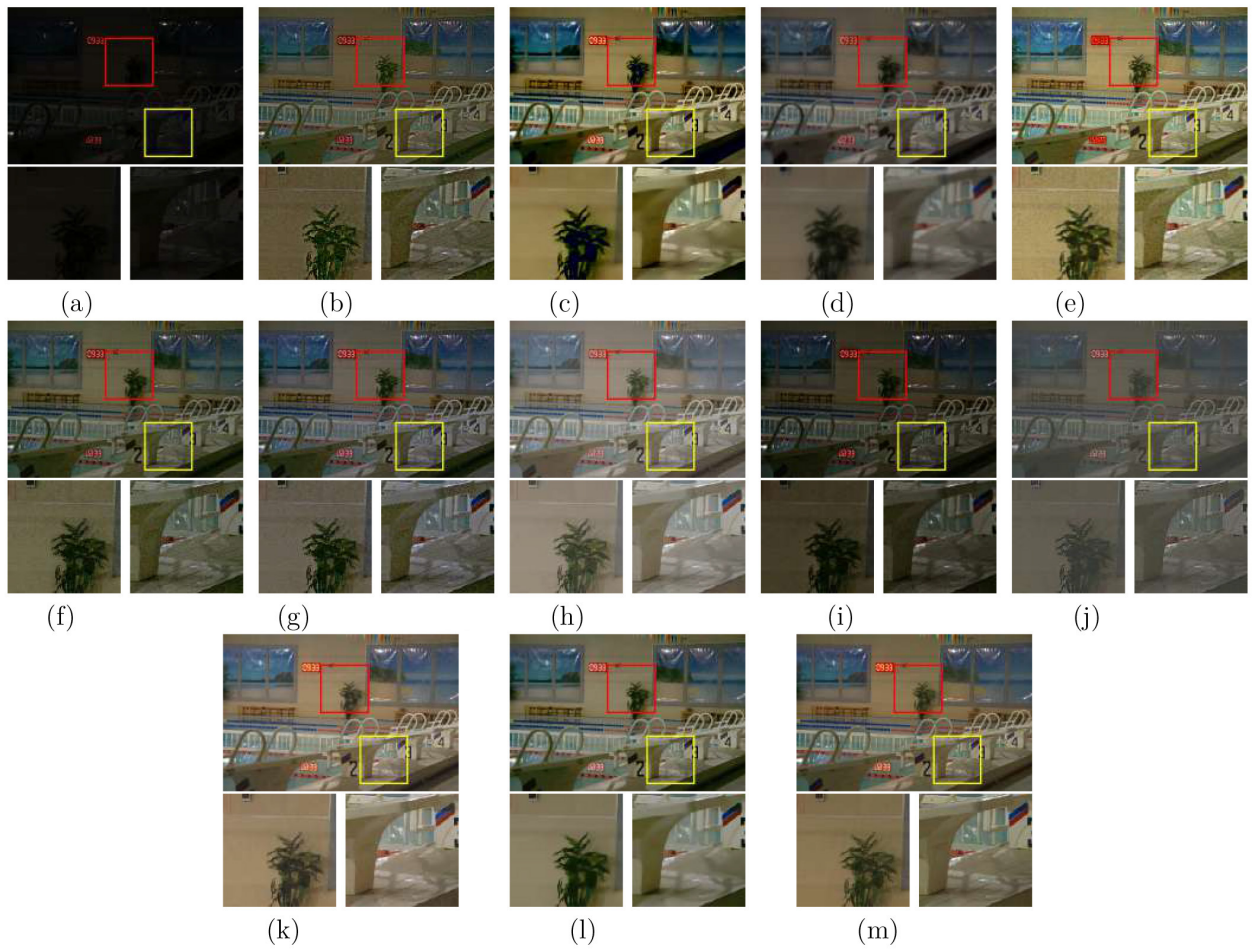
Qualitative comparison for Input-2 on the LOL dataset. Highlighted regions indicate zoomed-in comparisons for visual detail assessment. **(a)** Input-2, **(b)** LIME, **(c)** MBLLEN, **(d)** KIND, **(e)** SSIENeT, **(f)** Zero-DCE, **(g)** Zero-DCE++, **(h)** URetinex, **(i)** Brain, **(j)** NSGA, **(k)** LYT-Net, **(l)** Ours-2, **(m)** GT-2.

For the LOL dataset, image quality was evaluated using PSNR, SSIM, and LPIPS metrics, where higher PSNR and SSIM values and lower LPIPS values indicate better enhancement quality. The quantitative results are presented in Table 3, and qualitative comparisons are shown in Figures 1, 2.

**Table 3:** Quantitative performance and computational efficiency on the LOL dataset in terms of PSNR, SSIM, LPIPS, and average runtime per image (seconds).

Image No.	LOL			Run time in seconds
	PSNR↑	SSIM↑	LPIPS↓	
1	23.0898	0.8374	0.113	20.75
2	24.0145	0.7925	0.211	14.86
3	21.1841	0.6704	0.325	16.06
4	20.2598	0.7257	0.228	14.30
5	21.4539	0.8609	0.156	19.02
6	23.7748	0.7699	0.126	15.30
7	25.1065	0.8373	0.093	14.24
8	25.4082	0.9425	0.096	16.50
9	22.2945	0.7326	0.239	14.32
10	26.5870	0.8388	0.143	15.55
11	24.9413	0.7357	0.293	15.15
12	26.2953	0.8448	0.162	13.33
13	27.0905	0.9096	0.129	13.30
14	23.5258	0.7746	0.239	13.03
15	23.8224	0.8960	0.154	14.48
**Average**	**23.923**	**0.811**	**0.180**	**15.35**

Bold values indicate the average PSNR, SSIM, LPIPS, and runtime per image computed over all 15 test images in the LOL dataset.

**Figure 3:**
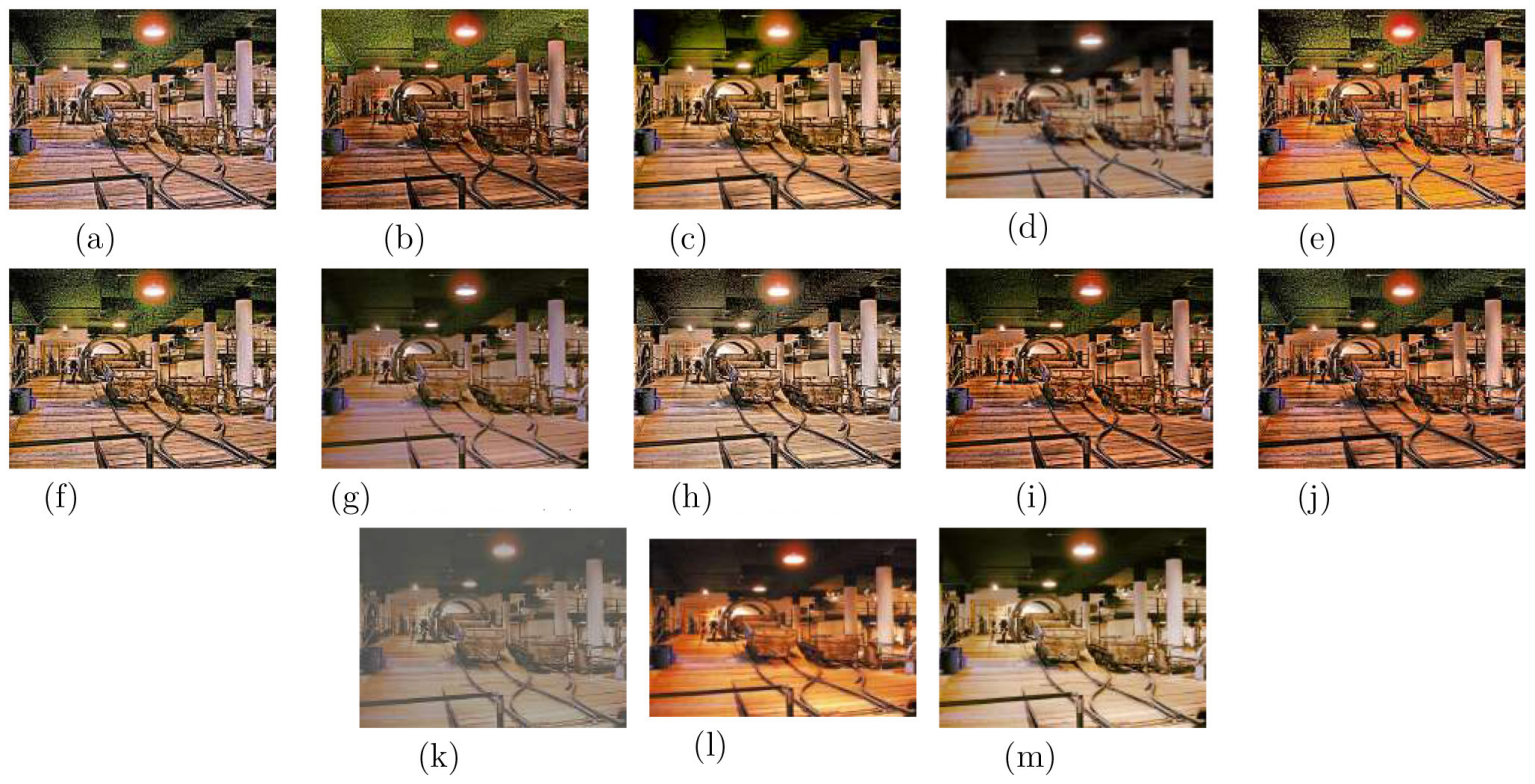
Qualitative results on the DICM low-light image dataset. **(a)** Input, **(b)** LIME, **(c)** MBLLEN, **(d)** KIND, **(e)** SSIENeT, **(f)** Zero-DCE, **(g)** Zero-DCE++, **(h)** URetinex, **(i)** Brain, **(j)** LightNeT, **(k)** NSGA, **(l)** LYT-Net, **(m)** Ours.

**Figure 4:**
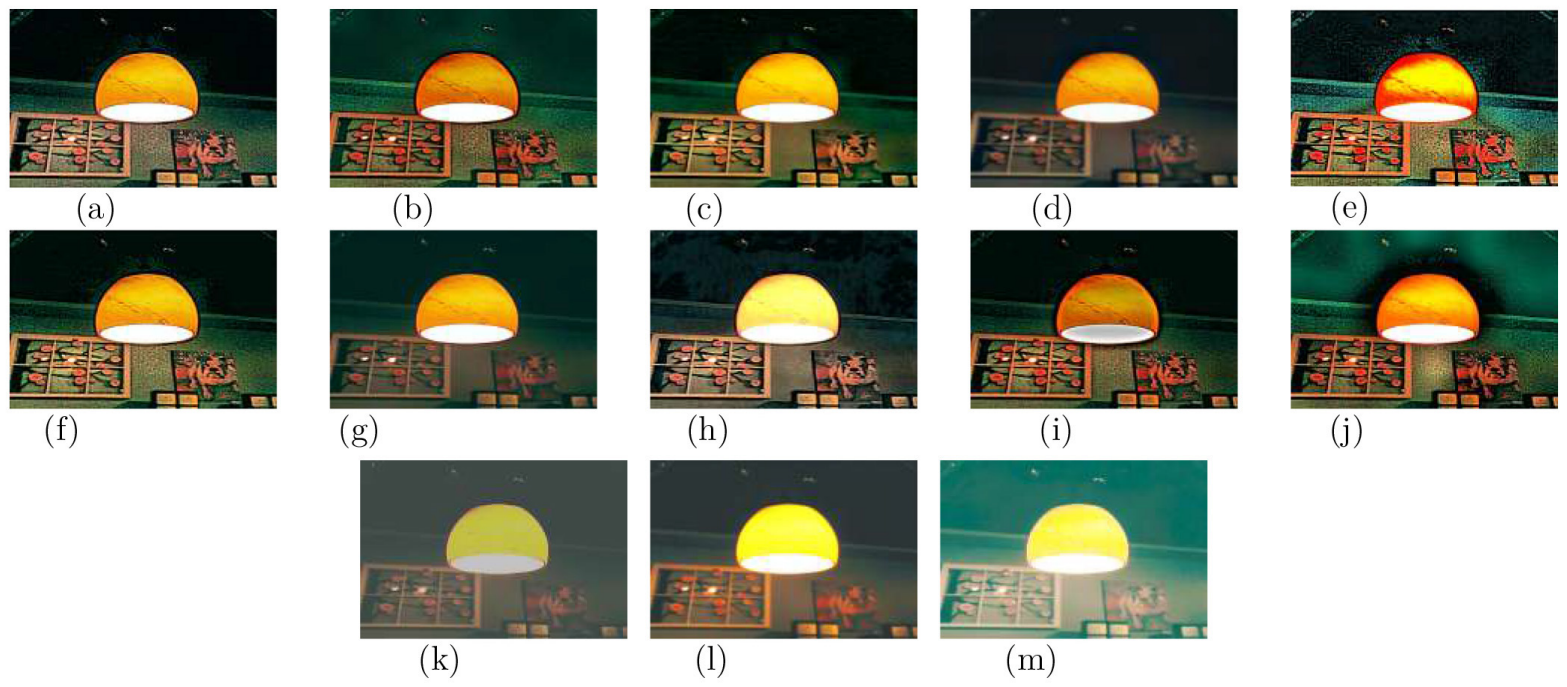
Qualitative results on the LIME low-light image dataset. **(a)** Input, **(b)** LIME, **(c)** MBLLEN, **(d)** KIND, **(e)** SSIENeT, **(f)** Zero-DCE, **(g)** Zero-DCE++, **(h)** URetinex, **(i)** Brain, **(j)** LightNeT, **(k)** NSGA, **(l)** LYT-Net, **(m)** Ours.

**Figure 5:**
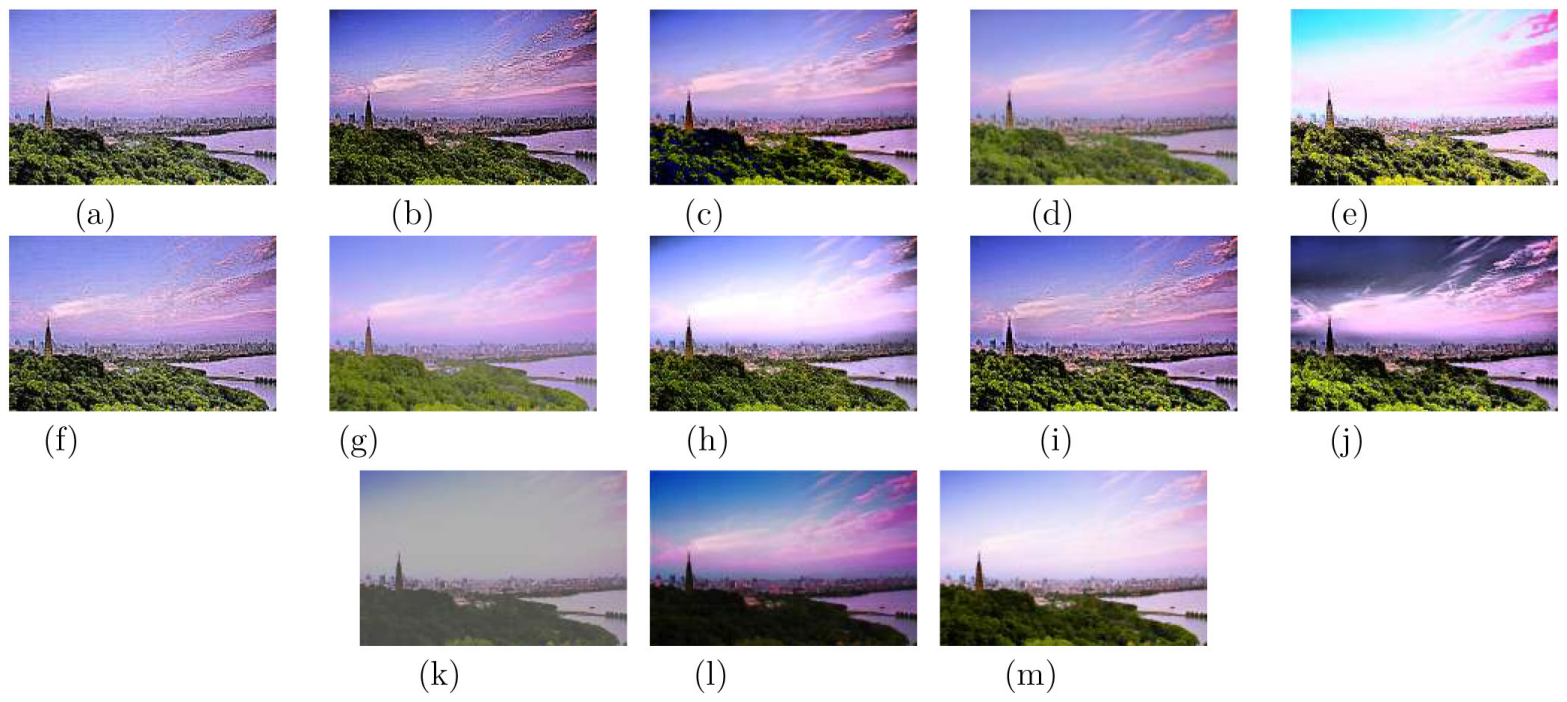
Qualitative results on the NPE low-light image dataset. **(a)** Input, **(b)** LIME, **(c)** MBLLEN, **(d)** KIND, **(e)** SSIENeT, **(f)** Zero-DCE, **(g)** Zero-DCE++, **(h)** URetinex, **(i)** Brain, **(j)** LightNeT, **(k)** NSGA, **(l)** LYT-Net, **(m)** Ours.

**Figure 6:**
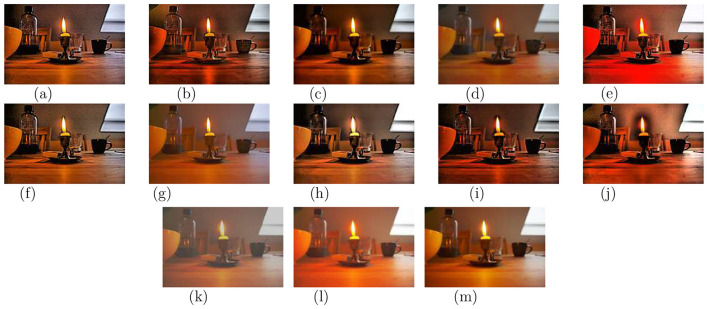
Qualitative results on the MEF low-light image dataset. **(a)** Input, **(b)** LIME, **(c)** MBLLEN, **(d)** KIND, **(e)** SSIENeT, **(f)** Zero-DCE, **(g)** Zero-DCE++, **(h)** URetinex, **(i)** Brain, **(j)** LightNeT, **(k)** NSGA, **(l)** LYT-Net, **(m)** Ours.

For the no-reference datasets (DICM, LIME, MEF, and NPE), image quality was assessed using NIQE and CEIQ metrics. Representative qualitative results are shown in Figures 3–6.

The proposed approach was compared with several existing methods, including LIME, Brain, KIND, MBLLEN, URetinex, LightNeT, SSIENeT, Zero-DCE, Zero-DCE++, NSGA, and LYT-Net. Quantitative comparisons on the LOL dataset are reported in Table 4, while the average results for the no-reference datasets are presented in Table 5. The obtained results show that the proposed framework performs effectively under different low-light conditions.

**Table 4:** Average quantitative comparison of low-light image enhancement methods on the LOL dataset in terms of PSNR, SSIM, and LPIPS.

Methods	LOL		
	PSNR↑	SSIM↑	LPIPS↓
LIME (Guo et al., 2016)	14.360	0.548	0.402
MBLLEN (Lv et al., 2018)	17.862	0.660	0.367
KIND (Zhang et al., 2019)	17.511	0.742	0.264
SSIENeT (Zhang et al., 2020)	18.362	0.662	0.391
Zero-DCE (Guo et al., 2020)	14.917	0.546	0.385
Zero-DCE++ (Li et al., 2021)	15.175	0.482	0.387
URetinex (Wu et al., 2022)	7.933	0.151	0.243
Brain (Cai and Chen, 2023)	11.459	0.423	0.359
LightNeT (Desai et al., 2023)	10.646	0.335	0.251
NSGA (Molina-Pérez and Rojas-López, 2024)	10.251	0.242	0.277
LYT-Net (Brateanu et al., 2025)	21.607	**0.811**	**0.134**
**Ours**	**23.923**	**0.811**	0.180

Best results are shown in bold, second-best results are underlined, and third-best results are highlighted in red.

**Table 5:** Average quantitative comparison on no-reference datasets (DICM, LIME, MEF, and NPE) in terms of NIQE, CEIQ, and Entropy.

Methods	DICM			LIME			MEF			NPE		
	NIQE↓	CEIQ↑	Entropy↑	NIQE↓	CEIQ↑	Entropy↑	NIQE↓	CEIQ↑	Entropy↑	NIQE↓	CEIQ↑	Entropy↑
LIME (Guo et al., 2016)	6.116	3.417	7.386	10.251	3.326	7.366	5.518	3.447	7.504	10.682	3.086	6.547
MBLLEN (Lv et al., 2018)	4.373	3.354	7.314	8.254	3.276	7.240	4.091	3.391	7.471	5.647	3.360	7.256
KIND (Zhang et al., 2019)	4.128	3.123	6.963	5.162	3.188	7.076	4.471	3.213	7.177	4.326	3.259	7.195
SSIENeT (Zhang et al., 2020)	6.574	3.296	7.251	8.288	3.269	7.117	5.632	3.289	7.168	6.968	3.258	7.101
Zero-DCE (Guo et al., 2020)	6.176	**3.460**	7.528	9.936	3.274	7.120	5.310	3.317	7.239	8.770	**3.454**	**7.428**
Zero-DCE++ (Li et al., 2021)	6.358	3.439	7.474	10.060	3.249	7.067	5.547	3.306	7.227	8.938	3.447	7.420
URetinex (Wu et al., 2022)	4.983	3.266	6.928	9.452	3.312	7.179	4.644	3.390	7.328	7.994	3.253	6.831
Brain (Cai and Chen, 2023)	5.065	3.250	7.134	8.426	3.170	7.055	4.629	3.234	7.220	6.043	3.326	7.160
LightNeT (Desai et al., 2023)	4.834	3.455	**7.537**	9.033	3.291	7.156	4.855	3.342	7.236	6.202	3.315	7.095
NSGA (Molina-Pérez and Rojas-López, 2024)	4.059	2.941	6.028	4.407	2.922	6.297	3.289	2.804	6.009	4.751	2.983	6.212
LYT-Net (Brateanu et al., 2025)	3.655	3.230	7.189	5.212	3.232	7.135	4.628	3.266	7.211	3.840	3.135	7.090
**Ours**	**3.187**	3.284	7.206	**4.164**	**3.503**	**7.499**	**3.549**	**3.489**	**7.525**	**3.565**	3.409	7.363

Best results are shown in bold, second-best results are underlined, and third-best results are highlighted in red.

### 5.1 Parameter sensitivity analysis

To study the influence of PSO parameters on enhancement performance, a sensitivity analysis was performed using different swarm sizes and iteration counts on the LOL test dataset. The average SSIM, PSNR, and runtime values were calculated over 15 test images.

As reported in Table 6, increasing the swarm size and the number of iterations produced only minor changes in SSIM and PSNR, while the computational time increased significantly. For instance, when the PSO configuration was changed from (*N* = 6, *T* = 8) to (*N* = 30, *T* = 100), the average SSIM showed only negligible variation (0.8112 vs. 0.8061), whereas the runtime increased considerably from 15.35 to 35.83 s.

**Table 6:** Sensitivity analysis of PSO swarm size and iteration settings on the LOL dataset.

Swarm Size (*N*)	Iterations (*T*)	Avg. SSIM	Avg. PSNR (dB)	Avg. Runtime (s)
6	8	0.8112	23.9231	15.35
10	30	0.8051	24.0189	21.91
20	50	0.8058	24.0416	34.51
25	70	0.8059	24.2549	35.70
30	100	0.8061	24.2404	35.83

Considering the balance between enhancement quality and computational efficiency, the configuration with *N* = 6 particles and *T* = 8 iterations was selected for the proposed framework. This compact setting provides stable enhancement performance with relatively lower runtime, indicating effective convergence of the optimization process. Since the proposed optimization involves only two parameters (α, γ) within a bounded search space, satisfactory convergence can be achieved using a smaller PSO configuration with reduced computational overhead.

Table 2 shows that moderate inertia weights provide better enhancement stability, whereas excessively large weights reduce optimization consistency. Similarly, smaller acceleration coefficients weaken optimization capability, while larger coefficients increase runtime with only marginal performance improvement. Therefore, *w* = 0.6, *c*_1_ = 1.3, and *c*_2_ = 1.3 were selected as balanced PSO parameters.

### 5.2 Stability analysis

To examine the stability of the proposed PSO-based optimization framework, experiments were conducted ten times on the LOL dataset using the same parameter configuration (*N* = 6, *T* = 8). Since PSO uses random particle initialization, repeated executions were carried out to observe possible variations in enhancement performance.

Table 7 reports the mean and standard deviation values of SSIM, PSNR, and runtime obtained from the ten independent runs. The relatively low standard deviation values indicate stable convergence and consistent enhancement performance across repeated experiments.

**Table 7:** Statistical stability analysis of the proposed method over ten independent runs on the LOL dataset.

Metric	Mean	Std. Dev.
SSIM	0.8001	0.0072
PSNR (dB)	23.6443	0.5202
Runtime (s)	14.8444	1.4015

### 5.3 Inferential statistical analysis

Table 8 presents the Wilcoxon signed-rank statistical analysis performed on the LOL dataset using SSIM and PSNR metrics. Most benchmark methods produced p-values smaller than the significance threshold of 0.05, indicating statistically significant differences between the proposed framework and existing enhancement methods. Although LYT-Net produced comparatively higher p-values, the proposed method still achieved competitive quantitative performance while maintaining stable enhancement quality. These results justify the trade-off between computational cost and enhancement performance of the proposed framework.

**Table 8:** Wilcoxon signed-rank statistical analysis on the LOL dataset using SSIM and PSNR metrics.

Method	SSIM *p*-value	PSNR *p*-value	Significant (*p* < 0.05)
LIME	6.10 × 10^−5^	6.10 × 10^−5^	Yes
MBLLEN	0.000122	6.10 × 10^−5^	Yes
KIND	0.01025	6.10 × 10^−5^	Yes
SSIENet	0.000305	0.000305	Yes
Zero-DCE	6.10 × 10^−5^	6.10 × 10^−5^	Yes
Zero-DCE++	6.10 × 10^−5^	0.000122	Yes
URetinex	6.10 × 10^−5^	6.10 × 10^−5^	Yes
Brain	6.10 × 10^−5^	6.10 × 10^−5^	Yes
LightNet	6.10 × 10^−5^	6.10 × 10^−5^	Yes
NSGA	6.10 × 10^−5^	6.10 × 10^−5^	Yes
LYT-Net	0.5994	0.0637	No

For LYT-Net, the obtained *p*-values are greater than 0.05, suggesting that both methods achieve statistically comparable performance on the LOL dataset. This observation is consistent with the quantitative evaluation results, where both methods demonstrate similar structural preservation capability.

Table 5 shows the entropy values obtained by all comparison methods on the DICM, LIME, MEF, and NPE datasets. In general, higher entropy values indicate richer image information and improved intensity variation after enhancement. The proposed method achieves competitive entropy results, highlighting the effectiveness of the PSO-based optimization process. However, entropy alone is not sufficient to guarantee visually pleasing enhancement, since excessive entropy optimization may also amplify noise or introduce artifacts. Therefore, additional no-reference BIQA metrics such as NIQE and CEIQ are considered to further evaluate perceptual quality, contrast improvement, and structural preservation, providing a more balanced assessment of the enhancement performance.

### 5.4 Results and discussion

The qualitative comparisons presented in Figures 1–6 demonstrate the performance of different enhancement methods under various low-light conditions. The results show noticeable differences in luminance recovery, texture preservation, edge clarity, and color consistency.

Figure 1 presents an indoor bowling scene with extremely poor visibility. LIME increases brightness but also amplifies noise and uneven illumination. Brain preserves moderate contrast, though several structures remain unclear. URetinex produces stronger enhancement but over-exposes brighter regions. SSIENeT introduces excessive whitening and saturation, while NSGA provides limited enhancement with reduced structural clarity. LYT-Net improves contrast but shows slight smoothing around lane boundaries. MBLLEN and Zero-DCE-based approaches improve visibility; however, texture degradation and smoothing artifacts are visible in the zoomed regions. In contrast, the proposed framework restores the bowling lanes, floor structures, and surrounding objects with balanced illumination and clearer structural details without severe saturation.

Figure 2 shows a challenging plant scene with heavily obscured foreground structures. LIME and Brain partially recover scene content but fail to restore fine details around the plant leaves and chair structures. URetinex produces excessive enhancement, resulting in a washed-out appearance, whereas SSIENeT introduces color distortion and reduced local contrast. NSGA provides moderate brightness improvement but insufficient recovery in darker regions. LYT-Net improves illumination and contrast; however, slight smoothing and texture loss remain visible. MBLLEN and Zero-DCE-based approaches improve brightness but still exhibit inconsistent contrast and reduced sharpness in textured areas. The proposed method restores the visibility of plant leaves, chair boundaries, and floor regions while maintaining consistent illumination and a more natural appearance. Slight attenuation of fine leaf textures can still be observed due to low-light noise suppression.

Figure 3 presents qualitative results on the DICM dataset with severe low-light degradation and visible noise. LIME produces uneven enhancement, while Brain retains dark background regions. URetinex causes over-enhancement near bright areas, and SSIENeT and MBLLEN introduce color shifts and excessive brightness. Zero-DCE and Zero-DCE++ improve visibility but retain haze-like artifacts and residual noise. NSGA provides limited enhancement, whereas LYT-Net improves illumination with slight smoothing in detailed regions. In contrast, the proposed framework achieves balanced luminance recovery, clearer structural visibility, and reduced noise amplification.

Figure 4 shows qualitative results on the LIME dataset containing localized bright light sources. Several methods excessively enhance the lamp region, causing saturation and loss of nearby details. URetinex and SSIENeT particularly produce strong over-exposure around the lamp boundary. MBLLEN and Zero-DCE-based methods improve brightness smoothly but reduce local contrast around the table structures. NSGA provides limited enhancement in darker regions, while LYT-Net introduces slight smoothing and color inconsistency near bright areas. The proposed method preserves the lamp boundary, surrounding objects, and table textures while maintaining balanced illumination without severe artifacts.

Figure 5 presents qualitative comparisons on the NPE outdoor scene with low visibility in cloud and vegetation regions. LIME and Brain provide limited enhancement in darker areas, while URetinex excessively enhances the sky region, causing unnatural brightness and loss of cloud details. SSIENeT introduces noticeable color distortion, whereas MBLLEN and Zero-DCE-based methods show smoothing and over-enhancement in brighter regions. NSGA provides insufficient enhancement, while LYT-Net slightly darkens some background regions. In contrast, the proposed framework preserves a more natural sky appearance with improved visibility and smoother brightness distribution.

Figure 6 shows qualitative results on the MEF dataset under strong illumination variation between the candle flame and surrounding dark regions. LIME and Brain slightly improve visibility but fail to recover darker objects effectively. URetinex causes saturation near the flame, while SSIENeT introduces reddish illumination and color imbalance. MBLLEN and Zero-DCE-based methods improve visibility but exhibit non-uniform brightness and reduced contrast. NSGA provides limited enhancement, whereas LYT-Net introduces slight over-brightness near the flame. The proposed framework provides balanced enhancement between bright and dark regions while preserving surrounding object visibility without severe saturation.

Overall, the qualitative comparisons show that the proposed framework improves low-light visibility while reducing over-exposure, color distortion, and noise amplification. BM3D preprocessing suppresses low-light noise before enhancement, while the intuitionistic fuzzy generator and PSO-based optimization provide adaptive enhancement under varying illumination conditions.

However, slight smoothing and attenuation of fine textures can still be observed in highly textured regions such as bowling pins, plant leaves, and background structures due to the denoising and non-linear enhancement stages.

**Figure 7:**
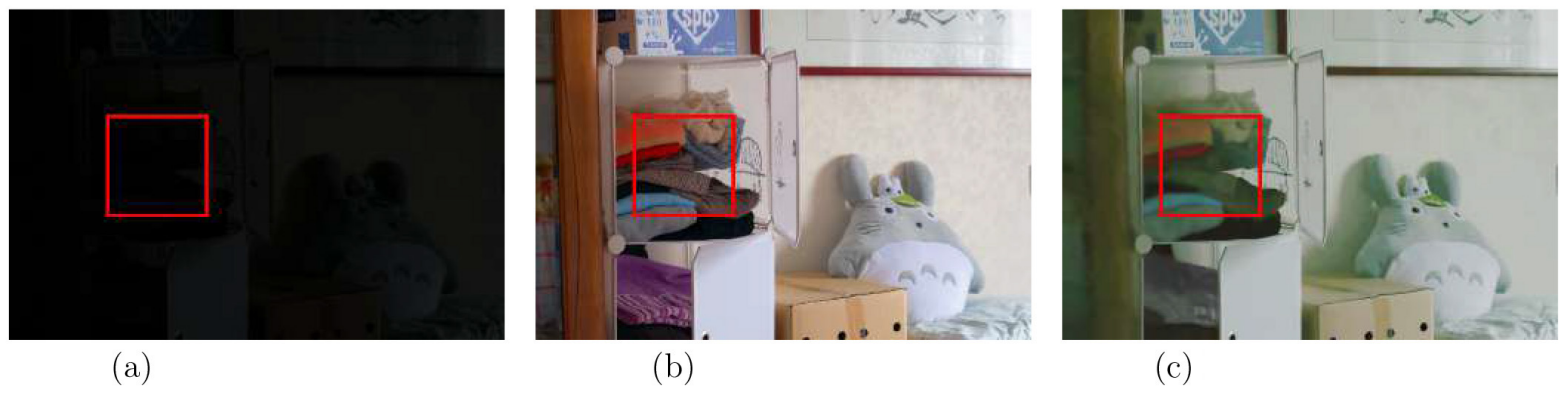
Failure case showing slight smoothing of fine textures. **(a)** Low-light Image, **(b)** Ground Truth, **(c)** Proposed Method.

As shown in Figure 7, slight smoothing of fine textures can be observed in the cloth region after enhancement. This behavior reflects the trade-off between noise suppression and preservation of high-frequency structural details under challenging low-light conditions.

Despite these limitations, the proposed framework maintains stable enhancement performance across multiple benchmark datasets while operating in a training-free manner without requiring paired training data or pre-trained models. The results indicate that the proposed image-driven optimization strategy remains effective under varying low-light conditions and diverse scene characteristics.

### 5.5 Ablation study

**Figure 8:**
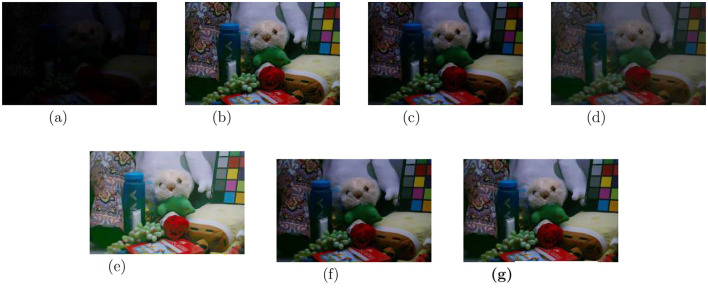
Visual comparison of ablation study components. From left to right: input image, without BM3D, without PSO, without IFG, without objective switching, without gamma correction, and the proposed method. **(a)** Input, **(b)** w/o BM3D, **(c)** w/o PSO, **(d)** w/o IFG, **(e)** w/o Objective switching, **(f)** w/o γ, **(g)** Proposed.

An ablation study was conducted on the LOL test dataset to evaluate the contribution of each component in the proposed framework. As shown in Table 9, removing BM3D reduces enhancement quality due to the presence of noise in low-light images. Specifically, the average SSIM decreases from 0.8112 to 0.6738 and the average PSNR decreases from 23.9231 dB to 21.1627 dB. The visual result in Figure 8b exhibits increased noise and reduced structural clarity, particularly in dark regions and textured areas, demonstrating the importance of effective noise suppression before enhancement. Excluding PSO also decreases performance, demonstrating the benefit of adaptive parameter optimization. As shown in Figure 8c, the resulting image exhibits slightly reduced contrast and detail visibility compared to the proposed method. The largest performance degradation occurs when the IFG component is removed. In this case, the average SSIM decreases from 0.8112 to 0.6019 and the average PSNR decreases from 23.9231 dB to 14.3163 dB. As observed in Figure 8d, the enhanced image exhibits noticeably lower contrast and poorer visibility of details in dark regions. This substantial reduction in both quantitative and visual quality highlights the critical role of IFG in contrast enhancement, brightness adjustment, and detail preservation. Replacing the proposed objective-switching strategy with a fixed entropy objective also lowers the enhancement performance, confirming the effectiveness of the adaptive objective-switching strategy for reference datasets. As shown in Figure 8e, the resulting image appears brighter but less natural, with reduced structural fidelity compared to the proposed method. Removing gamma correction results in only a moderate reduction in performance, suggesting that its primary contribution is brightness adjustment. The quantitative results in Table 9 and the visual comparisons in Figure 8 show that each component contributes to the enhancement process, while the complete framework achieves the best results.

**Table 9:** Average ablation study results on the LOL dataset.

Method	SSIM ↑	PSNR ↑	Time (s)
w/o BM3D	0.6738	21.1627	4.80
w/o PSO(fixed parameters)	0.6957	17.0102	10.99
w/o IFG	0.6019	14.3163	11.77
w/o Objective switching	0.7359	17.9417	13.62
w/o γ	0.7604	21.1822	10.68
**Proposed**	**0.8112**	**23.9231**	15.35

Bold values indicate the results obtained using the complete proposed method.

### 5.6 Computational efficiency

The computational efficiency of the proposed framework was assessed using the average processing time per image on the LOL test dataset, which contains paired low-light and reference images with representative image resolutions. The same enhancement procedure and parameter settings were applied to all no-reference datasets, including DICM, LIME, NPE, and MEF. All experiments were performed on a system equipped with an Intel i3-1005G1 CPU (1.20 GHz) and 4 GB RAM. As reported in Table 3, the proposed method requires an average processing time of 15.35 s per image for images with a resolution of 600 × 400 in the current MATLAB implementation.

Additional insight into the computational contribution of individual components is provided in Table 9, where all reported values represent average processing times per image. When the BM3D denoising stage is removed, the average processing time decreases from 15.35 s to 4.80 s. This observation indicates that BM3D preprocessing contributes approximately 10.55 s, corresponding to 69% of the total execution time, and therefore represents the most computationally demanding stage of the framework. The remaining 4.80 s is associated with the enhancement and optimization stages, including PSO-based parameter search, IFG computation, gamma correction, and fitness evaluation. Since these operations are repeatedly executed within each PSO iteration, their individual computational costs cannot be isolated precisely. Nevertheless, the results clearly demonstrate that BM3D denoising constitutes the primary computational bottleneck of the proposed approach.

Overall, the computational burden of the framework is mainly attributed to BM3D preprocessing and the repeated fitness evaluations performed during the iterative PSO optimization process. Although the proposed method requires more processing time than feed-forward deep learning approaches, it operates without any offline training stage and does not rely on large annotated datasets.

A runtime comparison with several representative low-light image enhancement methods is presented in Table 10. All reported runtimes were obtained by executing the publicly available implementations of the compared methods on the same hardware platform. As shown in Table 10, feed-forward learning-based methods such as Zero-DCE (0.95 s), Zero-DCE++ (0.63 s), and LYT-Net (2.81 s) achieve substantially faster runtimes than the proposed framework (15.35 s). This difference is primarily attributable to the BM3D denoising stage and the iterative PSO-based image-specific optimization procedure employed by the proposed framework. Nevertheless, the additional computational cost enables adaptive parameter tuning for each input image, making the framework particularly suitable for offline image enhancement tasks, scientific image analysis, and applications where interpretability and adaptability are more important than real-time execution.

**Table 10:** Average runtime comparison per image on the LOL dataset.

Method	Runtime (s)
Zero-DCE++	0.63
Zero-DCE	0.95
Brain	1.16
LYT-Net	2.81
MBLLEN	8.62
Ours	15.35

To further examine the balance between enhancement performance and computational cost, a Wilcoxon signed-rank statistical analysis was conducted (Table 8). The resulting p-values indicate statistically significant improvements in enhancement quality over most benchmark methods. Collectively, these findings demonstrate that the proposed framework provides a practical solution for offline and data-scarce low-light image enhancement scenarios, where adaptive and interpretable enhancement is preferred over black-box learning-based models.

### 5.7 Performance metrics

#### 5.7.1 Structural Similarity Index (SSIM)

The SSIM (Wang et al., 2004) between images *i* and *o* is defined in Equation (23) as:


$$\begin{array}{l} \text{SSIM}\left(i,o\right)=\frac{\left(2{\bar{\mu }}_{i}{\bar{\mu }}_{o}+c_{1}\right)\left(2\sigma_{io}+c_{2}\right)}{\left({\bar{\mu }}_{i}^{2}+{\bar{\mu }}_{o}^{2}+c_{1}\right)\left(\sigma_{i}^{2}+\sigma_{o}^{2}+c_{2}\right)}, \end{array}$$ (23)

where *c*_1_ and *c*_2_ are positive constants, and *i* and *o* represent the original and enhanced images, respectively; ${\bar{\mu }}_{i}$ and ${\bar{\mu }}_{o}$ denote the mean intensities of *i* and *o*, σ_*io*_ represents their covariance, and $\sigma_{i}^{2}$ and $\sigma_{o}^{2}$ are the variances of *i* and *o*, respectively.

#### 5.7.2 Peak Signal-to-Noise Ratio (PSNR)

PSNR (Dohmen et al., 2025) is a commonly used metric for evaluating the quality of a reconstructed image with respect to its original counterpart. It is expressed in decibels (dB) and is calculated from the mean squared error (MSE) between the corresponding pixels of the two images. A higher PSNR value indicates that the processed image is closer in quality to the original, implying less distortion and higher fidelity. PSNR is widely employed in image processing and compression to assess the effectiveness of enhancement and reconstruction algorithms. The PSNR is defined in Equation (24) as


$$\begin{array}{l} \text{PSNR}=10\cdot \log_{10}\left(\frac{\max{\left(I\right)}^{2}}{\text{MSE}}\right), \end{array}$$ (24)

where max(*I*) denotes the maximum possible pixel value of the image (e.g., 255 for 8-bit images). The MSE is defined in Equation (25) as


$$\begin{array}{l} \text{MSE}=\frac{1}{i\cdot j}\overset{i-1}{\underset{x=0}{\sum }}\overset{j-1}{\underset{y=0}{\sum }}{\left(I\left(x,y\right)-T\left(x,y\right)\right)}^{2}, \end{array}$$ (25)

where *I*(*x, y*) represents the pixel intensity at position (*x, y*) in the original image, and *T*(*x, y*) denotes the corresponding pixel intensity in the processed image.

#### 5.7.3 Learned Perceptual Image Patch Similarity (LPIPS)

LPIPS (Zhang et al., 2018) is a reference-based perceptual image quality metric that measures the similarity between two images using deep feature representations extracted from pretrained convolutional neural networks. The perceptual distance is computed by comparing feature activations across multiple network layers, with lower LPIPS values indicating higher perceptual similarity and better visual quality.

#### 5.7.4 Natural Image Quality Evaluator (NIQE)

NIQE (Mittal et al., 2012) is a no-reference image quality assessment metric that evaluates image quality based on deviations from natural scene statistics. It models the statistical regularities of natural images using a multivariate Gaussian model learned from high-quality undistorted images. The quality of a test image is quantified by measuring the statistical distance between its extracted features and the learned natural image model. Lower NIQE values indicate better perceptual quality and closer adherence to natural scene statistics.

#### 5.7.5 Contrast Enhancement Image Quality (CEIQ)

CEIQ (Fang et al., 2014) is a no-reference image quality assessment metric that evaluates the effectiveness of contrast enhancement by extracting structural and entropy-based statistical features and mapping them to perceptual quality scores using a regression model.

### 5.8 Limitations

Although the proposed framework achieves effective low-light image enhancement, certain limitations remain. The integration of BM3D denoising and PSO-based IFG optimization increases computational complexity, limiting real-time applicability in the current MATLAB implementation. In some cases, slight smoothing of fine textures may occur in low-contrast regions. Additionally, since SSIM is directly used as the optimization objective for the LOL dataset, SSIM-based comparisons may be less independent. Therefore, complementary metrics such as PSNR and LPIPS, along with qualitative visual analysis, are also considered for evaluation. Future work will focus on improving computational efficiency and preserving fine structural details through hybrid fuzzy and lightweight deep learning approaches.

## 6 Conclusion

This paper presented an intuitionistic fuzzy generator (IFG)-based framework for low-light image enhancement that combines BM3D denoising with PSO-based adaptive gamma correction. Experimental results on both reference and no-reference datasets show that the proposed method effectively improves contrast, preserves important structural details, and reduces noise under challenging low-light conditions. The framework also achieves competitive performance when compared with several state-of-the-art enhancement methods. In addition, the sensitivity analysis and statistical stability study demonstrate the robustness and consistency of the PSO-based optimization process under different parameter settings.

Despite these advantages, the current framework has a relatively high computational cost, which limits its suitability for real-time applications. Future work will therefore focus on reducing computational complexity and improving the preservation of fine local details. This can be achieved by developing hybrid approaches that combine the proposed fuzzy framework with lightweight deep learning models to enable faster, more efficient, and robust low-light image enhancement.

## Data Availability

Publicly available datasets were analyzed in this study. This data can be found here: https://doi.org/10.6084/m9.figshare.27192921.
